# Effects of Using a Special Weighted Vest on Muscle Activity around the Scapula during Knee Push-Up Plus in Healthy Subjects

**DOI:** 10.3390/healthcare12171778

**Published:** 2024-09-05

**Authors:** Lin Liu, Ying Liu, Young-sam Yuk, Hyoung-won Lim

**Affiliations:** 1 Department of Physical Therapy, Graduate School, Dankook University, Cheonan 31116, Republic of Korea; lin282870@gmail.com (L.L.); liuying7051426@gmail.com (Y.L.); 2 Liberal Arts College, Dankook University, Cheonan 31116, Republic of Korea; 3 Department of Physical Therapy, College of Health Sciences, Dankook University, Cheonan 31116, Republic of Korea

**Keywords:** scapular dyskinesia, push-ups plus, muscle activity, weighted vest, serratus anterior

## Abstract

Muscle imbalances in the upper body can lead to ineffective movement patterns and potential injury. The purpose of this study was to investigate the muscle activity, impact, and muscle activation ratio of the serratus anterior (SA), upper trapezius (UT), lower trapezius (LT), and pectoralis major (PM) during the knee push-up plus (KPUP) exercise under various loads. Method: Electromyography assessed scapular muscle activity in 32 healthy adults (15 males, 17 females) during three KPUP variations. Results: PM and UT showed no significant activity differences across loads, whereas SA and LT did. SA activity was significantly higher in the weighted KPUP (WKPUP) 3 kg than that in KPUP and WKPUP 1 kg. LT activity was also significantly higher in WKPUP 3 kg compared to KPUP and WKPUP 1 kg, with KPUP showing higher activity than WKPUP 1 kg. PM/SA ratios remained consistent across loads, while UT/LT ratios varied significantly, being notably lower at 3 kg compared to 0 kg and 1 kg. Similarly, UT/SA ratios differed significantly among loads, being notably lower at 3 kg and 1 kg compared to 0 kg. Conclusion: WKPUP with 3 kg demonstrated significantly higher SA and LT activity compared to KPUP and WKPUP 1 kg. The lowest UT/LT ratio was observed during the WKPUP 3 kg, suggesting its effectiveness for optimizing muscle activation balance during KPUP exercises. These findings may inform the development of exercise protocols aimed at improving scapular stabilization.

## 1. Introduction

Musculoskeletal shoulder pain can lead to significant pain and functional impairment, affecting work, hobbies, social and sporting activities, and potentially leading to psychological distress and a reduced quality of life [1]. Subacromial impingement syndrome (SIS) is the most common diagnosis in patients with shoulder pain, accounting for about 44–65% of cases [2]. It is primarily caused by mechanical issues and anatomical abnormalities, leading to shoulder pain and impaired function [3,4].

The serratus anterior (SA) and lower trapezius (LT) muscle activity serves to stabilize the scapula against the chest wall and is essential for scapular upward rotation and retraction [5]. Push-up plus (PUP) exercise is very effective in activating the SA and reducing upper trapezius (UT) activation [6]. In previous studies, researchers proposed various PUP exercises to enhance SA muscle activation [7,8]. Batbayar et al. found that the SA muscle showed increased activation when using a wider shoulder-width hand position during PUP exercises [9]. Clinically, push-up plus exercises in the kneeling position (KPUP) are recommended for female patients to reduce physical demands [10,11]. Ludewig et al. [12] measured the UT to SA activity ratio during modified PUP exercises and found a significant reduction in UT/SA ratio, emphasizing effective targeting of imbalanced muscles. A high pectoralis major (PM)/SA ratio has been linked to shoulder joint pathology [13], underscoring the importance of examining the PM/SA ratio during SA-activating exercises.

The weighted vest (WV) is often used as a tool to increase body load, manipulate warm-up intensity, increase bone density, and improve long jump ability and explosive power [14,15]. Utilizing a weighted vest in long-term interventions, including strength training, walking, stair climbing, and balance exercises, effectively improves muscle power, strength, physical functioning, bone density, weight loss, pain reduction, and lateral stability in sedentary individuals and older adults [16,17,18]. A previous study revealed that utilizing a WV (20% of body weight) during push-ups increased muscle strength, enabling advanced weightlifters to achieve maximum strength [19]. That resistance exercise for 9 months with a WV (5–20% of body weight) improved muscle strength and balance in older adults compared to a conventional activity control group [20]. These studies suggest that weighted vests may be useful for increasing muscle strength.

In a previous study, muscle activity and activity ratio of the SA and UT muscles were examined during bilateral arm elevation with varying loads (no load, 1 kg, and 3 kg) [21]. This study reported increased muscle activity in UT, LT, and SA muscles for both SIS and No-SIS groups. However, the load was applied using dumbbells in both hands rather than directly targeting scapular retraction. While qualitative evidence suggests increased muscle activity with this load application method, no study has specifically investigated muscle activity and activity ratio with direct loading for scapular retraction.

Therefore, in this study, we aimed to determine the muscle activity and muscle activation ratio of the muscles around the scapula (SA, UT, and LT) and PM by applying a load only to the scapula during the KPUP exercise, which is known to effectively activate the SA muscle. We hypothesized that the weighted KPUP (WKPUP) (3 kg) condition might exhibit higher SA muscle activity compared to both the WKPUP (1 kg) and the no-load conditions. Additionally, we expected that the UT/SA, UT/LT, and PM/SA muscle activity ratios in the WKPUP (3 kg) condition might be lower than those in the no-load and WKPUP (1 kg) conditions.

## 2. Materials and Methods

### 2.1. Participants

The sample size was determined using a medium effect size of 0.25, a statistical significance level of 0.05, and a high statistical power of 0.80, showing that having 28 subjects was sufficient. To account for potential dropouts, we recruited 4 additional participants, resulting in a total of 32 subjects (15 male and 17 female) aged between 21 and 32 years. All subjects gave voluntary informed consent before participating in the study, which was approved by Dankook University’s Institutional Review Board (IRB No. 2023-10-019-006). The participants were chosen based on the following inclusion criteria: (1) no instability or musculoskeletal issues in the neck, shoulder, or upper extremities, (2) no shoulder muscle strengthening exercises in the last 3 months, (3) no neurological issues, and (4) no shoulder surgery in the previous 6 months. Subjects who experienced pain during maximum voluntary isometric contraction (MVIC) were excluded from the study.

### 2.2. Electromyographic Recording and Data Processing

EMG signals were recorded using the Desk DTS EMG system to measure surface electromyography. Muscle activity during KPUP, WKPUP (1 kg), and WKPUP (3 kg) exercises was compared. Data analysis was conducted using Noraxon MyoResearch 3.6, with a sampling rate of 1000 Hz. A band-pass filter ranging from 20 Hz to 450 Hz was applied to the collected data. The raw data underwent processing to compute the root mean square (RMS) with a 50 ms window. EMG signals were normalized using maximum voluntary isometric contraction (MVIC). Muscle activity was measured by determining the electrode attachment sites for each muscle, informed by previous research [22,23]. The skin was lightly sanded and then sterilized with alcohol to prepare the electrode attachment areas. If needed, the subjects’ hair was shaved. Surface electrodes were positioned at specific sites on the body to measure muscle activity in the UT, LT, SA, and PM muscles of the subjects. The electrodes were attached to the upper extremity of each subject’s dominant side, which was consistently the right side for all participants. Surface EMG recordings were obtained for the UT, LT, SA, and PM muscles, and the subjects performed standardized manual muscle-testing positions to determine the MVIC of each muscle. The subjects maintained each test position for 5 s, and, to reduce prolonged muscle exhaustion that might arise during MVIC assessments, the subjects rested for 1 min after each measurement. Each muscle’s MVIC was measured three times. After processing the RMS data for 5 s, the average EMG signal volume for 3 s, excluding the initial and final seconds, was utilized to determine muscle activity. Correct electrode placement was confirmed by visual inspection of the EMG signals on a computer screen during specific muscle testing (%MVIC).

### 2.3. Procedures

Before starting each exercise, the participants were introduced to the KPUP, WKPUP (1 kg), and WKPUP (3 kg) conditions, were given a detailed explanation for five minutes to familiarize them with the KPUP, and received a demonstration from the researcher.

**Figure 1:**
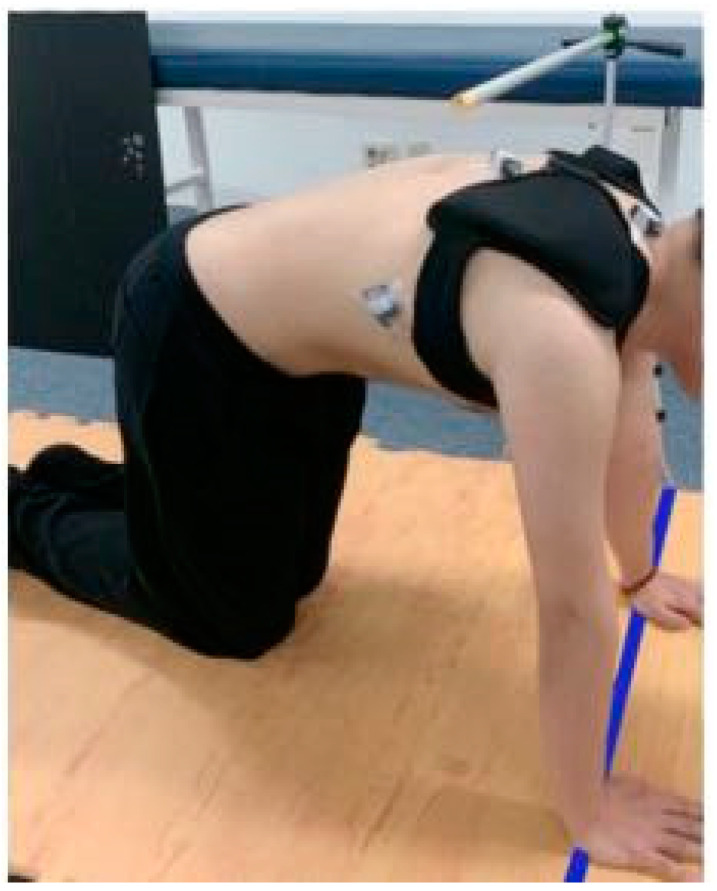
Weighted knee push-up plus.

All subjects performed three postures randomly to exclude order effects. The KPUP exercise was performed for three trials in a quadruped position, with hands shoulder-width apart, hips and knees bent at 90 degrees, and knees also shoulder-width apart. To standardize exercise posture and performance across the three conditions and control unnecessary movements, the phases of the exercise were operationally defined as follows: (a) starting phase: protracting the scapula by moving the chest backward until the thoracic spinous process touches the target bar for 2 s, (b) holding phase: maintaining the posture for 5 s, (c) ending phase: returning to the starting position for 2 s. A one-minute rest was taken between trials to minimize muscle fatigue. During the holding phase of each trial, EMG signals were collected for an intervening 3 s. The average of the three trials per KPUP exercise was analyzed, and data were omitted if the standardized starting phase, holding phase, or ending phase were not completed correctly. All tests were performed in a standardized quadruped position. The target bar was positioned at the T-4 spinous process level so that the subjects could protract the scapula to the same height and maintain a consistent posture for each KPUP trial [23]. To prevent excessive forward movement, a target line was placed in a palm-supported position. The duration of each exercise was controlled using auditory signals from a metronome. KPUP was considered successful when the subject’s T-4 spinous process reached the target bar and both shoulders remained in place without crossing the target line (Figure 1).

### 2.4. Weighted Vest (WV)

**Figure 2:**
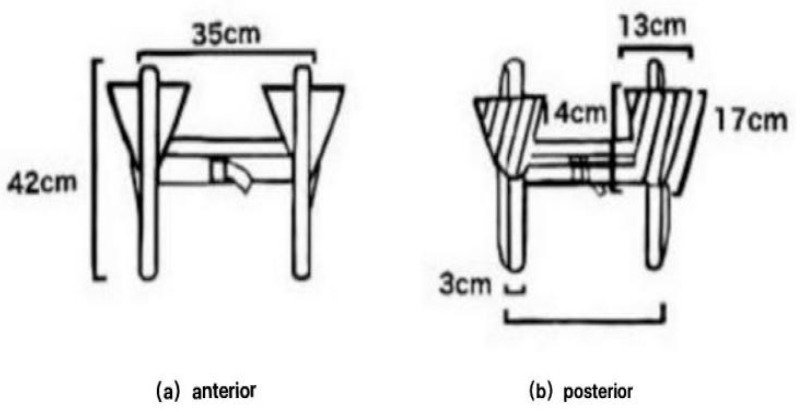
Design drawing of special weighted vest.

This study used a traditional weighted vest modified specifically for this research. The load-bearing part is an inverted triangle similar in size and shape to the scapula, with narrow shoulder straps placed to minimize the impact on EMG collection and reduce interference with electrode attachment. The vest comprises two shoulder straps, two inverted triangle bags, and an adjustable strap. The shoulder straps connect at the chest, while the inverted triangle bags rest on both sides of the back, matching the shoulder blades. Each bag measures 14 cm in height, 13 cm at the base, 17 cm at the hypotenuse, and 2 cm in thickness. Made of nylon cloth and filled with 1 kg and 3 kg iron balls in the pockets, these bags provide the necessary weight required (Figure 2).

### 2.5. Statistical Analysis

Statistical analysis was conducted using SPSS Statistics version 29.0 (IBM, Armonk, NY, USA). Normalized EMG values were calculated as means ± standard deviations, expressed as a percentage of MVIC. The Shapiro–Wilk test was used to confirm the normal distribution of the continuous variables, and all outcome variables were found to be normally distributed. To evaluate the variances among the three conditions (no load, 1 kg, and 3 kg) on muscle activity (SA, UT, LT, and PM) and ratios (PM/SA, UT/SA, and UT/LT) during the KPUP exercise, a one-way repeated ANOVA was performed. Pairwise comparisons were conducted using Bonferroni’s post hoc analysis to further analyze the effects of different loads on LT, UT, PM, and SA, with a significance level set at α = 0.05 for all statistical tests.

## 3. Results

### 3.1. General Characteristics of the Participants

The descriptive characteristics of the study subjects are presented in Table 1.

### 3.2. Comparison of Muscle Activity during KPUP, WKPUP (1 kg), and WKPUP (3 kg)

There was no difference in muscle activity between the PM and UT muscles across the different loads (*p* > 0.05). However, differences were observed in the SA and LT muscles. Post hoc analysis revealed that the muscle activity of the SA at 3 kg was significantly greater than that found at 0 kg and 1 kg (*p* < 0.05), with no significant difference between 1 kg and 0 kg (*p* > 0.05). LT muscle activity was also significantly higher during WKPUP at 3 kg compared to KPUP and WKPUP at 1 kg, with KPUP showing higher activity than WKPUP at 1 kg (Table 2).

### 3.3. Comparison of Scapular Stabilizer Muscle Activity Ratios during KPUP, WKPUP (1 kg), and WKPUP (3 kg)

The PM/SA ratios remained consistent across loads (*p* > 0.05). However, the UT/LT ratios varied significantly (*p* < 0.05), being noticeably lower at 3 kg compared to 0 kg and 1 kg. Similarly, the UT/SA ratios differed significantly among loads (*p* < 0.05), being noticeably lower at 3 kg and 1 kg compared to 0 kg (Table 3).

## 4. Discussion

SA plays a critical role in maintaining proper scapula positioning and motion, in preventing shoulder impingement and instability, and in reducing the risk of musculoskeletal disorders in patients with shoulder and neck pain [24]. To the best of our knowledge, this is the first study to enhance SA muscle activation during KPUP exercises using a weighted vest designed to load only the scapula.

The results showed that different loading conditions alter the levels of muscle activity and muscle activity ratios. The main finding is that, during the WKPUP (3 kg) exercise, the SA and LT muscle activities were significantly higher than those observed during KPUP and WKPUP (1 kg). Additionally, during the WKPUP (3 kg) condition, the UT/LT and UT/SA ratios were significantly lower compared to the 0 kg condition. Thus, these findings substantiate our hypothesis. However, the PM/SA ratio decreased as the weight increased to 1 kg and 3 kg compared to the no-load condition, but this difference was not statistically significant. This suggests that the hypothesis predicting lower PM/SA muscle activity ratios in the WKPUP (3 kg) condition compared to both the no-load and WKPUP (1 kg) conditions was not supported. Interestingly, the WKPUP (3 kg) condition did not increase PM activity, despite allowing more weight on the upper extremity, which could potentially improve scapular stability and thereby enable selective SA activation.

Maenhout et al. [25] investigated muscle activity in KPUP and its variants to identify exercises that correct intramuscular imbalance between UT and SA by increasing SA activity and lowering the UT/SA ratio. Their results showed that heterolateral leg extension during KPUP increased LT activity, while homolateral leg extension increased SA activity. In their results, SA activity was 44.20%, whereas, in our study, SA activity was higher, at 49.44% in the 3 kg condition. Possible explanations for these results include the possibility that homolateral leg extension places more weight on the upper extremities, increasing SA activity, and that heterolateral leg extension increases LT activity by activating the gluteus maximus and tightening the thoracolumbar fascia [25]. Another study compared the muscle activity of the scapular stabilizers during KPUP and modified Vojta’s three-point support (MV3PS) exercises, finding that SA was selectively activated in MV3PS [26]. This increased activation is due to the lower hand position in MV3PS and the concentration of weight on the elbow and shoulder joints during the plus phase, which increases the demand on the shoulder muscles. Similar results were observed in our study. Weighted vests designed to load the scapula increase the demand on the shoulder muscles. Specifically, the WKPUP (3 kg) condition likely increased muscle activity by providing resistance to the SA muscle, the primary mover during the plus phase.

Excessive PM activity increases anterior translation of the humerus, causing joint instability [27]. Another study reported that a standardized PUP, with less scapular protraction due to decreased SA activity, increased PM activity to achieve the same range of motion through additional clavicle protraction and humeral translation [23]. DeMay et al. [28] found that SA activity decreased and PM activity increased when performing a knee prone bridging exercise on an unstable Redcord sling (RS). They concluded that the PM was the only muscle with significantly increased activity due to the RS. Previous research has proposed that an unstable base of support leads to greater muscle activation in the proximal muscles [29]. Therefore, because an unstable surface was not used in this study, the compensatory action of the PM would have been minimized.

In our study, there were no significant differences in UT among the three load conditions. Excessive UT activity is a characteristic of individuals with weak SA, which puts them at higher risk of developing impingement syndrome and shoulder pain [30]. Ludewig et al. [12] investigated the effects of different push-up variations (KPUP, wall PUP, and standard PUP) on the SA and UT. Their results showed that the standard PUP had the highest SA activity and LT/SA ratio during the plus phase. Kang et al. [6] reported in their meta-analysis of SA and UT EMG during the PUP exercise that performing the PUP exercise on an unstable surface may induce higher levels of UT activation without increasing SA activation. They suggested that, if the goal of the exercise program is to strengthen the SA muscles with less UT activity, PUP exercises should be performed on a stable surface. They found that using a shoulder flexion angle of 110° or 120°, performing the PUP exercise with fully extended elbows, and lifting the ipsilateral lower limb led to higher SA and lower UT EMG activity [6]. Andersen et al. [31] found that only the press-up and PUP exercises activated the lower trapezius and serratus anterior more than the upper trapezius. Based on the results mentioned above, our study demonstrated that the optimal activation of the SA muscle could be similarly achieved by applying weight to the upper extremity, akin to lifting the lower extremity at a standard shoulder width and minimizing UT activation on a stable surface.

In our study, LT muscle activity was significantly higher at 3 kg compared to both no load and 1 kg. Patients with shoulder pain or instability often have higher UT activity and lower LT and SA activity [32]. Ludewig et al. [12] proposed that exercises involving a protraction component significantly affect SA activation, noting that exercises like PUP result in a significantly lower UT/SA ratio. B. Horsak et al. [10] reported a small increase in LT activity when training was performed on a stable support base. Adding load increases muscle recruitment and differentiates muscle patterns during elevation exercises [33]. Therefore, it is believed that LT activity increased under the conditions of WKPUP (3 kg) with a stable support base and an additional load.

Cools et al. [34] suggested that excessive UT activity combined with reduced middle or lower trapezius activity may contribute to abnormal scapular motion, leading to subacromial impingement syndrome. Patients with subacromial impingement syndrome reported a higher UT/LT and a lower LT/SA ratio during active arm lifting compared to healthy controls [35]. D. Kara et al. [36] recommended scapular retraction exercises at 0° shoulder abduction in early rehabilitation to minimize UT/MT and UT/LT ratios. While their study used a standing position, ours utilized a quadruped position with 0° shoulder abduction and fully extended elbows. Despite the differences in posture, our study induced scapular retraction using a target line and bar, minimizing abnormal compensatory trapezius muscle activity. Therefore, it is believed that the UT/LT and UT/SA ratios in the WKPUP (3 kg) condition were significantly lower than those observed in the 0 kg condition. Ludewig et al. [12] proposed that exercises with a protraction component, such as PUP, significantly activate the SA and lower the UT/SA ratio. A low UT/SA ratio means that SA is highly activated relative to UT being minimally activated [12]. For SA strengthening programs requiring maximal SA activation and a low UT/SA ratio, the KPUP exercise with a weighted vest appears optimal compared to other modified PUP exercises.

This study had several limitations. First, we only assessed the activities of the SA, UT, LT, and PM, neglecting other muscles that may influence scapular control, such as the rhomboids, levator scapulae, and middle trapezius. Second, our sample consisted of relatively young participants (21–32 years of age). Third, scapular kinematics were not assessed. Fourth, individuals with clinical conditions such as shoulder pain, scapular winging, and subacromial impingement syndrome were excluded from the study. Future research should consider conducting additional studies focusing on individuals with shoulder pathologies such as scapular winging and subacromial impingement syndrome, taking into account the above limitations.

## 5. Conclusions

This study investigated the knee push-up plus exercise with specific emphasis on loading the scapula, leading to the following key findings: The WKPUP (3 kg) exercise showed higher muscle activity in SA and LT compared to KPUP and WKPUP (1 kg). Additionally, the UT/LT ratio was noticeably lower in the WKPUP (3 kg) condition. Therefore, the WKPUP (3 kg) exercise is recommended as the optimal exercise for healthy subjects when maximum SA activation and a minimum UT/LT ratio are required. Furthermore, the use of a weighted vest may enhance the balance of activation between the scapular stabilizing muscles during KPUP exercises. Future research should explore the application of this exercise in different populations, such as in individuals with shoulder pathologies, and investigate the long-term effects of incorporating weighted vests into rehabilitation or strength training programs.

## Figures and Tables

**Table 1:** General characteristics of Subjects (n = 32).

Gender	Age (Years)	Height (cm)	Weight (kg)
Male (15)	27.00 ± 2.5	175.33 ± 6.1	73.83 ± 13.4
Female (17)	25.58 ± 2.3	164.64 ± 5.5	56.32 ± 6.9

**Table 2:** Comparison of muscle activity (% MVIC) during KPUP, WKPUP (1 kg), and WKPUP (3 kg) (n = 32).

Variable	0 kg	1 kg	3 kg	F	*p*
UT	12.32 ± 5.94	11.73 ± 6.13	13.11 ± 6.50 ^b^	3.057	0.054
PM	12.39 ± 6.83	11.86 ± 6.03	14.27 ± 7.95 ^b^	3.062	0.073
SA	36.66 ± 18.19	40.53 ± 16.65	49.44 ± 20.93 ^ab^	17.224	<0.001 *
LT	6.64 ± 3.03	5.52 ± 2.92 ^a^	8.81 ± 3.55 ^ab^	23.993	<0.001 *

PM: pectoralis major; LT: lower trapezius; SA: serratus anterior; UT: upper trapezius; ^a^: significantly different from the 0 kg group (*p* < 0.05); ^b^: significantly different from the 1 kg group (*p* < 0.05) in post hoc Bonferroni analysis; * *p* < 0.05.

**Table 3:** Comparison of scapular stabilizer muscle activity ratios during KPUP, WKPUP (1 kg), and WKPUP (3 kg) (n = 32).

Variable	0 kg	1 kg	3 kg	Z	*p*
	Effect Estimate (MD, 95% CI)				
UT/SA	0.30 (0.23, 0.53)	0.25 (0.17, 0.49) ^a^	0.25 (0.16, 0.40) ^a^	13.938	<0.001 *
UT/LT	1.83 (1.03, 3.19)	2.31 (1.16, 4.01)	1.69 (0.89, 2.40) ^ab^	20.313	<0.001 *
PM/SA	0.38 (0.19, 0.67)	0.24 (0.16, 0.52)	0.28 (0.14, 0.50)	2.688	0.261

UT/SA: upper trapezius/serratus anterior ratio; UT/LT: upper trapezius/lower trapezius ratio; PM/SA: pectoralis major/serratus anterior ratio; ^a^: significantly different from the 0 kg group in post hoc Bonferroni analysis (*p* < 0.05); ^b^: significantly different from the 1 kg group in post hoc Bonferroni analysis (*p* < 0.05); * *p* < 0.05.

## Data Availability

Original data are available from the corresponding author upon reasonable request.
